# Zebrafish cutaneous injury models reveal that Langerhans cells engulf axonal debris in adult epidermis

**DOI:** 10.1242/dmm.049911

**Published:** 2023-04-03

**Authors:** Eric Peterman, Elgene J. A. Quitevis, Erik C. Black, Emma C. Horton, Rune L. Aelmore, Ethan White, Alvaro Sagasti, Jeffrey P. Rasmussen

**Affiliations:** ^1^ Department of Biology, University of Washington, Seattle, WA 98195, USA; ^2^ Molecular and Cellular Biology Program, University of Washington, Seattle, WA 98195, USA; ^3^ Molecular, Cell and Developmental Biology Department, University of California, Los Angeles, CA 90095, USA; ^4^ Molecular Biology Institute, University of California, Los Angeles, CA 90095, USA; ^5^ Institute for Stem Cell and Regenerative Medicine, University of Washington, Seattle, WA 98109, USA

**Keywords:** Zebrafish, Wallerian degeneration, Homeostasis, Tissue repair, Wound healing, Somatosensory axons

## Abstract

Somatosensory neurons extend enormous peripheral axons to the skin, where they detect diverse environmental stimuli. Somatosensory peripheral axons are easily damaged due to their small caliber and superficial location. Axonal damage results in Wallerian degeneration, creating vast quantities of cellular debris that phagocytes must remove to maintain organ homeostasis. The cellular mechanisms that ensure efficient clearance of axon debris from stratified adult skin are unknown. Here, we established zebrafish scales as a tractable model to study axon degeneration in the adult epidermis. Using this system, we demonstrated that skin-resident immune cells known as Langerhans cells engulf the majority of axon debris. In contrast to immature skin, adult keratinocytes did not significantly contribute to debris removal, even in animals lacking Langerhans cells. Our study establishes a powerful new model for studying Wallerian degeneration and identifies a new function for Langerhans cells in maintenance of adult skin homeostasis following injury. These findings have important implications for pathologies that trigger somatosensory axon degeneration.

## Teaser

**Summary:** We introduce new models for studying axon degeneration with high spatiotemporal resolution, revealing that skin-resident immune cells known as Langerhans cells engulf axonal debris in zebrafish skin.

## INTRODUCTION

Skin is a dynamic organ that constantly replenishes its constituent cells during homeostasis and in response to injury. Skin provides protection to environmental insults by functioning both as a durable barrier and sensory organ. Dense networks of somatosensory axon endings arborize throughout the epidermis, the outermost layer of the skin, and detect a variety of stimuli, including pain, temperature and itch (Handler and Ginty, 2021). Cutaneous injuries and wounds damage these fragile axons, which can trigger Wallerian degeneration (WD), a molecular program of axon degeneration (Coleman and Höke, 2020). WD leaves the neuronal soma intact, but the axon distal to the injury degenerates. WD creates a myriad of axonal debris fragments that must be removed to restore full functionality to the skin. This presents a particular challenge for the skin, given the enormous size and complexity of cutaneous arbors (Wu et al., 2012).

Phagocytic cells engulf and degrade axon debris following WD, which allows for potential axon reinnervation and promotes tissue homeostasis by preventing inflammation. Typically, peripheral nerve damage triggers recruitment of professional phagocytes derived from the immune system, which infiltrate the distal nerve and phagocytose cellular debris (Msheik et al., 2022; Zigmond and Echevarria, 2019). For example, CX3CR1^+^ dermal nerve-associated macrophages scan nerves following injury, engulf myelin and promote reinnervation (Kolter et al., 2019).

Surprisingly, studies in the larval skin of *Drosophila melanogaster* and *Danio rerio* revealed that, rather than relying on macrophages or other immune cells, epidermal keratinocytes engulf and degrade nearly all cutaneous somatosensory neurite debris (Han et al., 2014; Rasmussen et al., 2015). A limitation of these models is that the larval epidermis of these animals contains only a monolayer or bilayer of keratinocytes and lacks the diverse repertoire of immune cell types that appear later in vertebrate skin organogenesis (Botting and Haniffa, 2020). Thus, whether these models accurately reflect the cellular and molecular mechanisms involved in removal of axonal debris in mature, stratified skin remains unknown. Identification of the phagocytes involved in debris removal in the adult epidermis is relevant for understanding post-embryonic pathologies in which axon homeostasis is altered, such as diabetic and chemotherapy-induced peripheral neuropathy (Stucky and Mikesell, 2021).

Here, we develop *ex vivo* and *in vivo* models to assess somatosensory axon degeneration and subsequent phagocytosis in adult zebrafish scales. Our approach allows live-cell imaging of WD in the presence of all resident cell types found in the adult vertebrate epidermis, including stratified keratinocytes and diverse immune cell types. By imaging axon degeneration and the associated cellular responses, we identify the cells responsible for axon debris clearance following degeneration in adult epidermis. In contrast to larval animals, epidermal keratinocytes do not play a major role in debris engulfment. Rather, we find that Langerhans cells, a skin-resident immune cell type mainly studied for their antigen-presenting roles (Doebel et al., 2017; Kaplan, 2017), engulf the majority of cutaneous axon debris. Notably, keratinocytes do not engulf increased quantities of axonal debris in the absence of Langerhans cells. Altogether, our work establishes scale explants as a tractable system to study WD, allowing us to image this process in a stereotypical fashion with high spatiotemporal resolution. We specifically highlight the cell biology of injury responses in the adult skin and reveal that larval and adult skin use different mechanisms for debris removal.

## RESULTS

### The adult zebrafish scale as a model for WD

**Fig. 1.**
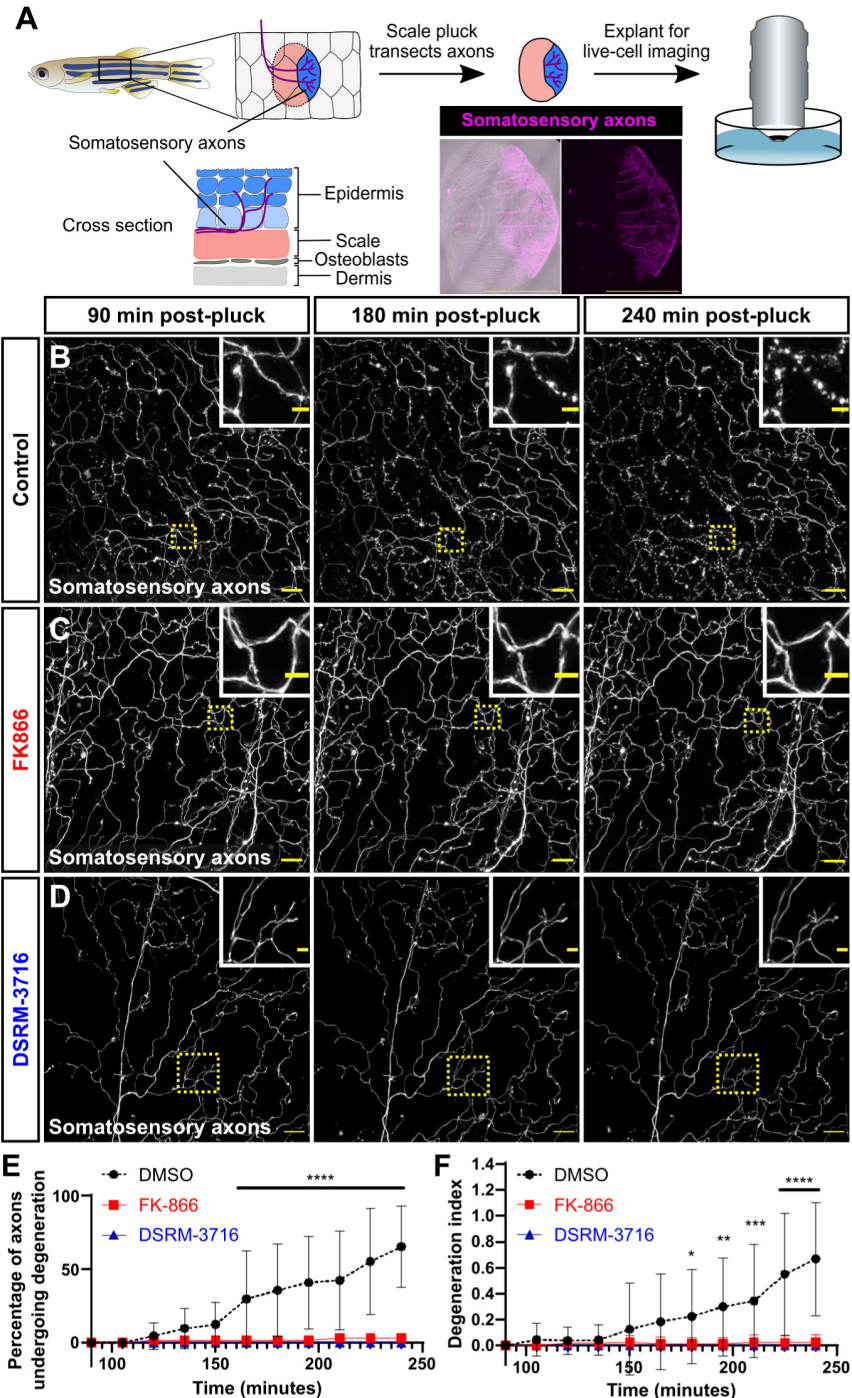
***Ex vivo* scale explants as a model for Wallerian degeneration.** (A) Schematic depicting the anatomy of the adult zebrafish scale epidermis and scale removal. The stratified epidermis (above the bony scale) is innervated by the peripheral axons of dorsal root ganglion somatosensory neurons. Confocal image shows an example of an entire scale explant expressing a somatosensory axon reporter [magenta; *Tg(p2rx3a:mCherry)*]. (B-D) Confocal images of time-lapses of explanted scales from adults expressing a somatosensory axon reporter [*Tg(p2rx3a:mCherry)*]. Scales were treated with DMSO as a vehicle control (B), FK866 (C) or DSRM-3716 (D). Dotted line boxes denote the regions magnified in insets. Note the lack of axon degeneration in the FK866- and DSRM-3716-treated scales. See Movie 1. (E,F) Percentage of axons undergoing degeneration (E) and the axon degeneration index (F) in control, FK866-treated and DSRM-3716-treated scales. *n*=8 for control, *n*=12 for FK866, *n*=8 for DSRM-3716 [regions of interest (ROIs)]. Two-way ANOVA followed by Bonferroni tests determined significance of differences between control, FK866 and DSRM-3716. *P<0.05, **P<0.01, ***P<0.001, ****P<0.0001. Scale bars: 1 mm (A), 20 μm (B-D), 5 μm (B-D, insets). Error bars in E and F represent s.d.

In order to follow the fate of degenerating cutaneous axons in adult skin, we sought a simple method to trigger WD of somatosensory axons. We previously demonstrated that the peripheral axons of dorsal root ganglion somatosensory neurons densely innervate the epidermis above adult scales (Rasmussen et al., 2018). Scales are dermal appendages that cover the adult trunk in an overlapping pattern akin to tiles on a roof (Fig. 1A). Physically removing (‘plucking’) scales from adult fish removes both the bony scale and attached epidermis with resident cell types including keratinocytes and peripheral axons. Scale removal severs cutaneous axons from their somata (Fig. 1A). To determine whether axons degenerate within explanted scales, we plucked and cultured scales from adults expressing a reporter for a subset of somatosensory neurons [*Tg(p2rx3a:lexA;LexAOP:mCherry)*, hereafter referred to as *Tg(p2rx3a:mCherry)* (Palanca et al., 2013)]. Using live-cell imaging to monitor axon degeneration in real time (Fig. 1A), we found that approximately half of the mCherry^+^ axons initiated degeneration between 165 and 240 min post-pluck, generating significant amounts of axon debris in the epidermis (Fig. 1B,E,F; Movie 1).

To determine whether the axon degeneration we observed was specifically due to WD, we incubated scales in two different small-molecule inhibitors of WD. First, we used FK866, an inhibitor of nicotinamide phosphoribosyltransferase (Nampt). FK866 has previously been shown to inhibit WD in culture and following axotomy of larval somatosensory neurons (Di Stefano et al., 2015). Second, we used DSRM-3716, a sterile-α and Toll/interleukin 1 receptor motif containing protein 1 (Sarm1) antagonist (Hughes et al., 2021). SARM1 is necessary for the normal progression of WD (Osterloh et al., 2012). We found that, compared to vehicle treatment, treating explanted scales with either of these inhibitors potently blocked axon degeneration, up to 4 h post-pluck (Fig. 1C-F; Movie 1). Thus, explanted scales can serve as a simple model of WD in the adult epidermis. Furthermore, inhibition of Nampt or Sarm1 after axon severing is sufficient to inhibit WD.

### Creation of transgenic tools to monitor keratinocyte phagosomes in adult epidermis

What are the cell(s) responsible for engulfing axon debris following WD in adult skin? We previously demonstrated that together the two layers of larval keratinocytes (periderm and basal cells) engulf essentially all cutaneous axon debris (Rasmussen et al., 2015). Similarly, keratinocyte-like epidermal cells internalize and degrade neurite debris in larval *Drosophila* skin (Han et al., 2014). Thus, we began by examining keratinocyte contributions to debris removal in adult skin. The epidermis stratifies during post-larval growth, adding layers of suprabasal cells in between the periderm and basal cell layers (Guzman et al., 2013; Rangel-Huerta et al., 2021). In adult scales, somatosensory axons arborize throughout basal and suprabasal epidermal layers, often in direct contact with keratinocytes (Rasmussen et al., 2018).

To track the phagocytic ability of adult keratinocytes by live-cell imaging, we expressed EGFP-2xFYVE, which binds to a phospholipid enriched in early and late phagosome membranes (Gillooly et al., 2000), in two overlapping keratinocyte populations. First, we used upstream regulatory sequences from the keratinocyte marker *krt4* to drive expression of EGFP-2xFYVE [*Tg(krt4:EGFP-2xFYVE)*]. Second, we engineered a bacterial artificial chromosome (BAC) with EGFP-2xFYVE in place of the start codon of *ΔNp63* [*TgBAC(ΔNp63:EGFP-2xFYVE)*]. Using laser axotomy to induce WD in larval trigeminal neurons, we confirmed that these EGFP-2xFYVE^+^ compartments colocalized with axonal debris after WD (Fig. S1A). In adult epidermis, we found that *Tg(krt4:EGFP-2xFYVE)* labeled spherical, phagosome-like compartments within keratinocytes predominantly in the periderm layer (Fig. S1B,C), while *TgBAC(ΔNp63:EGFP-2xFYVE)* labeled similar structures of basal and suprabasal keratinocytes (Fig. S1B,D). We further validated that these transgenes label a subset of adult keratinocyte phagosomes by demonstrating that the EGFP-2xFYVE^+^ structures colocalized with LysoTracker, a live-cell dye that labels acidic compartments (Fig. S1E). Together, these new tools allow for the unambiguous tracking of keratinocyte phagocytosis at larval and adult stages.

### Keratinocytes do not play a significant role in debris engulfment following axon degeneration

**Fig. 2.**
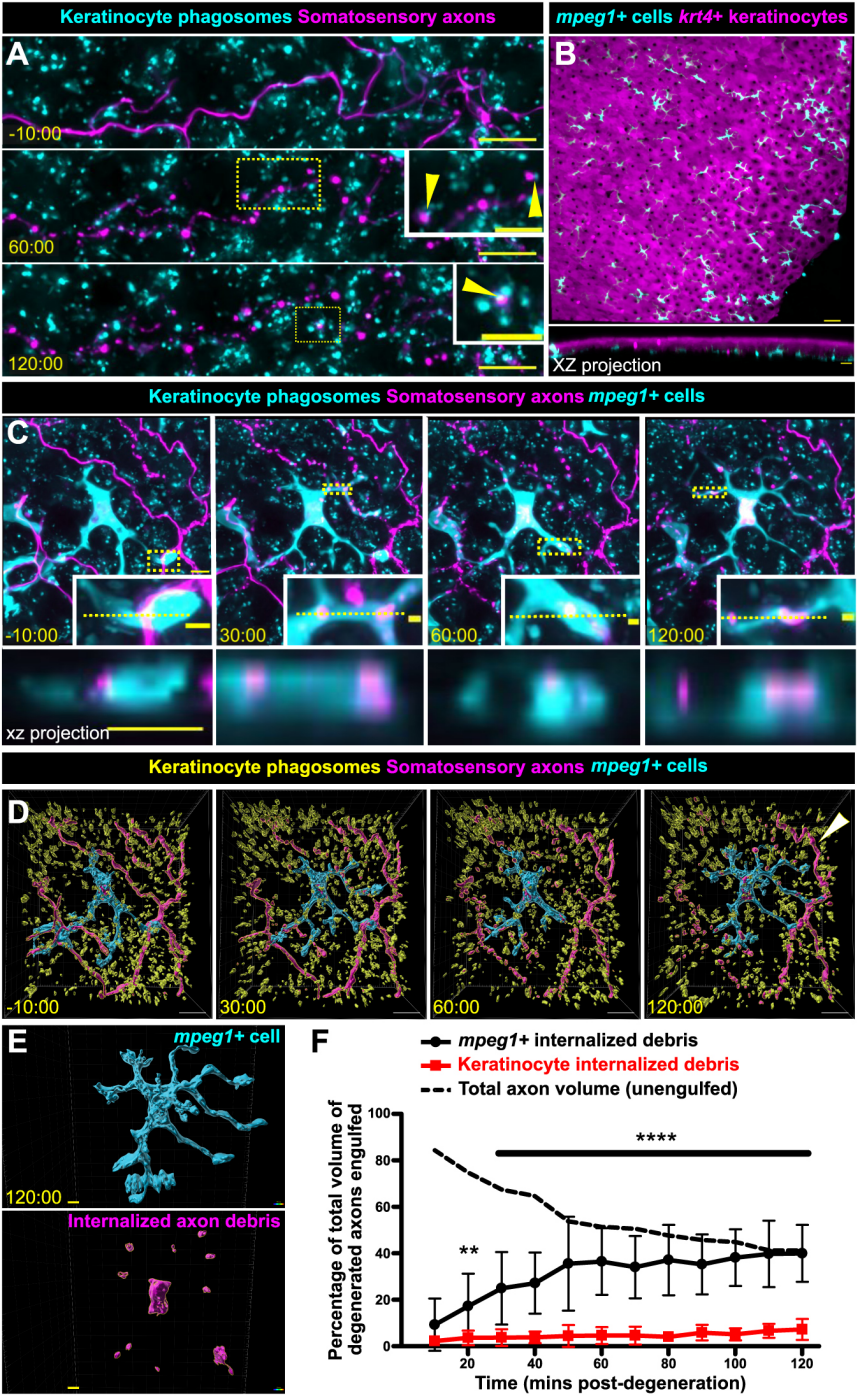
***mpeg1^+^* cells, not keratinocytes, engulf the majority of axon debris following axon degeneration.** (A) Confocal images from a time-lapse of scale pluck-induced axon degeneration from an adult expressing reporters for keratinocyte phagosomes [*Tg(krt4:EGFP-2xFYVE);TgBAC(ΔNp63:EGFP-2xFYVE)*] and somatosensory axons [*Tg(p2rx3a:mCherry)*]. Arrowheads show examples of colocalization. Time denotes mm:ss relative to the onset of axon degeneration. See Movie 2. (B) Lateral confocal image (top) and reconstructed cross-section (bottom), showing that *mpeg1^+^* cells (cyan) densely populate the scale epidermis and reside beneath the *krt4*^+^ layer (magenta). (C) Confocal images from a time-lapse of axon degeneration from an adult expressing reporters for keratinocyte phagosomes [*Tg(krt4:EGFP-2xFYVE);TgBAC(ΔNp63:EGFP-2xFYVE)*], somatosensory axons [*Tg(p2rx3a:mCherry)*] and *mpeg1^+^* cells [*Tg(mpeg1:NTR-EYFP)*] before and during scale pluck-induced axon degeneration. Yellow dotted lines in insets denote the plane reconstructed in the *xz* projections. Time denotes mm:ss relative to the onset of axon degeneration. See Movies 3 and 4. (D) Surface views from Imaris of the panels in C showing keratinocyte phagosomes (yellow), *mpeg1^+^* cell (cyan) and somatosensory axons (magenta). Arrowhead indicates intact axon. (E) Surface view used in Imaris from C (120:00) for volume-engulfed quantifications. (F) Quantification of total axon volume engulfed over time by keratinocytes and *mpeg1^+^* cells. Two-way ANOVA followed by Bonferroni tests determined significance of differences between *mpeg1^+^* cells and keratinocytes. **P<0.01, ****P<0.0001. *n*=10-16 cells/ROIs from *n*=12 scales from *N*=9 fish. Scale bars: 5 μm [A, insets, C, C (*xz* projection), E], 10 μm (A,D), 30 μm (B), 2 μm (C, insets). Error bars in F represent s.d.

To assess keratinocyte involvement in axon debris clearance in adult epidermis, we live imaged scale explants with axons labeled with mCherry and *krt4^+^*/*ΔNp63^+^* keratinocyte phagosomes labeled with EGFP-2xFYVE. The relative pH stability of mCherry (Cranfill et al., 2016) allowed us to track axon debris over extended periods of time. We found that, similar to larval keratinocytes, adult keratinocytes could internalize axon debris (Fig. 2A, arrowheads). However, adult keratinocytes did not significantly contribute to debris removal, engulfing less than 10% of axonal debris after WD (Fig. 2A,F; Movie 2). These results suggest that axon debris either remains largely unengulfed, or a different phagocytic cell type clears axon debris in adults.

### *mpeg1*^+^ cells engulf large quantities of debris following axon degeneration

Because keratinocytes did not engulf appreciable amounts of axonal debris, we questioned whether other cell types clear axonal debris in adult epidermis. Previous reports suggest that a variety of immune cells populate the skin throughout organogenesis (Kasheta et al., 2017; Kuil et al., 2020; Lin et al., 2019; Lugo-Villarino et al., 2010; Wittamer et al., 2011); therefore, we reasoned that the adult epidermis would have a larger variety of immune cells present to possibly participate in debris engulfment. Using cell type-specific transgenic animals, we found that the adult scale epidermis contained *mpeg1^+^* (also known as *mpeg1.1*^+^) Langerhans cells and metaphocytes, *lck^+^* lymphocytes and *mpx^+^* neutrophils (Fig. 2B; Fig. S2).

Owing to the high density of *mpeg1^+^* cells and their roles in repair and phagocytosis in other contexts (Casano et al., 2016; Peri and Nüsslein-Volhard, 2008; Petrie et al., 2014; Rosenberg et al., 2012), we postulated they may participate in axon debris engulfment. To directly compare the relative contributions of *mpeg1^+^* cells and keratinocytes to debris removal, we created quadruple transgenic animals expressing reporters for *mpeg1^+^* cells, *krt4^+^*/*ΔNp63^+^* keratinocyte phagosomes, and axons. By live-cell imaging axon degeneration over a period of hours, we found that *mpeg1^+^* cells engulfed debris at a significantly higher efficiency than that of keratinocytes (Fig. 2C-F; Movies 3 and 4). Orthogonal cross-sections revealed that debris was fully internalized within *mpeg1^+^* cells, which frequently relied on dynamic protrusions to internalize the debris (Fig. 2C). Notably, debris engulfment plateaued 2 h post-axon degeneration (Fig. 2F). To determine whether the debris not internalized by *mpeg1*^+^ cells was taken up by other cells or remained engulfed, we stained scales with LysoTracker at 2 h post-axon degeneration and found that the persistent debris failed to stain positive for LysoTracker (Fig. S3), suggesting that it remained unengulfed.

**Fig. 3.**
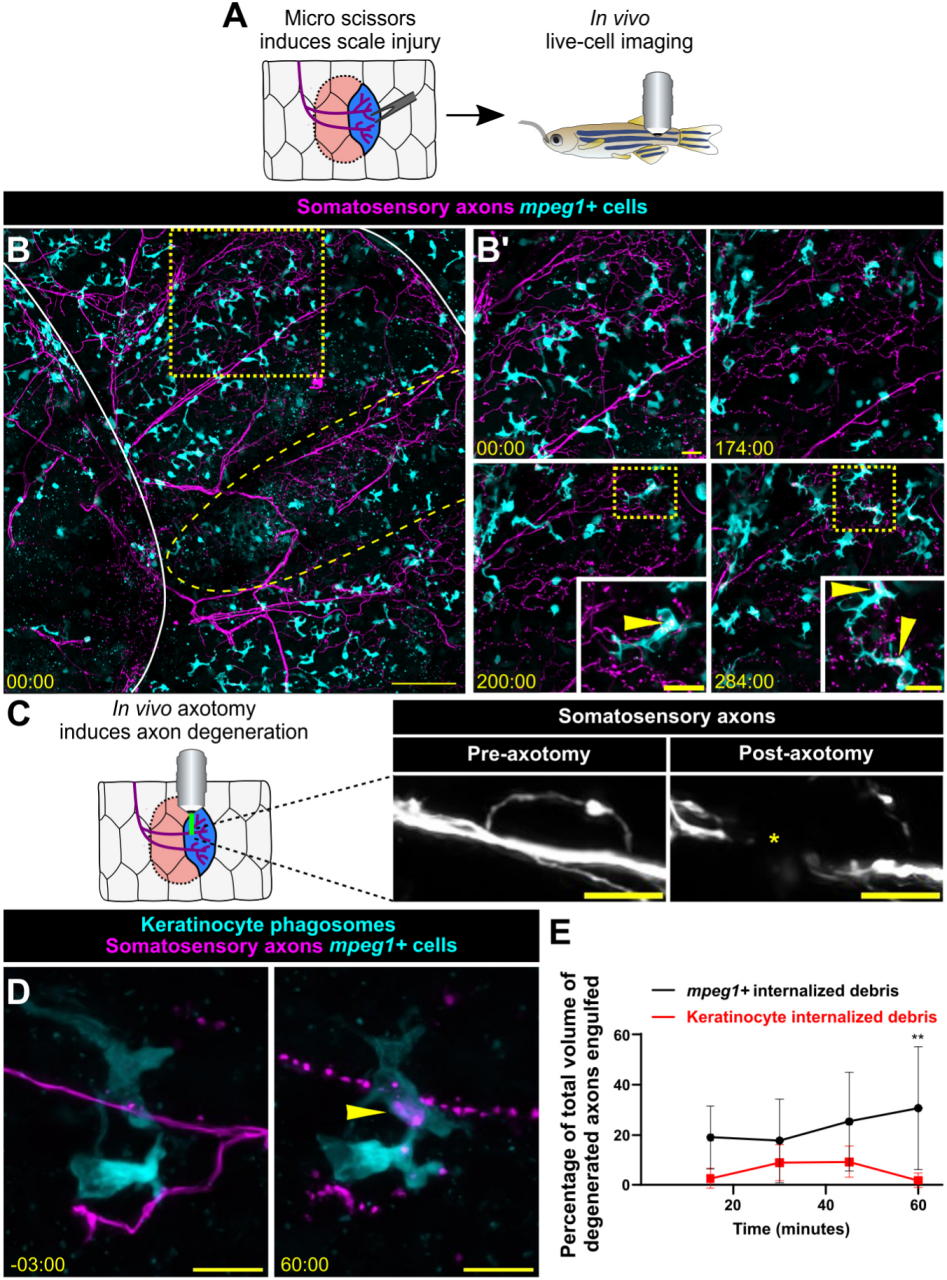
***mpeg1^+^* cells engulf axonal debris after cutaneous injury *in vivo*.** (A) Schematic for inducing scale injury via micro scissors *in vivo*. (B) Confocal images from a time-lapse of *in vivo* micro scissor scale injury from an adult expressing reporters for somatosensory axons [*Tg(p2rx3a:mCherry)*] and *mpeg1^+^* cells [*Tg(mpeg1:NTR-EYFP)*]. Solid white lines outline scales from fish, yellow dashed line oval denotes site of micro scissor injury, yellow dotted line box denotes the region magnified in B′. Yellow dotted line boxes in B′ denote the regions magnified in insets. Time denotes mm:ss relative to the time of injury. See Movies 5 and 6. (C) Schematic for laser axotomy *in vivo*. Representative image of axons pre- (left) and post- (right) laser axotomy. Yellow asterisk denotes site of axotomy. (D) Confocal images from a time-lapse of axon degeneration from an adult expressing reporters for keratinocyte phagosomes [*Tg(krt4:EGFP-2xFYVE);TgBAC(ΔNp63:EGFP-2xFYVE)*], somatosensory axons [*Tg(p2rx3a:mCherry)*] and *mpeg1^+^* cells [*Tg(mpeg1:NTR-EYFP)*] before and during laser axotomy-induced axon degeneration. Arrowhead denotes engulfed axonal debris. (E) Quantification of total axon volume engulfed over time by keratinocytes and *mpeg1+* cells. Two-way ANOVA followed by Bonferroni tests determined significance of differences between *mpeg1^+^* cells and keratinocytes. **P<0.01. *n*=8 ROIs from *N*=3 fish. Scale bars: 100 μm (B), 20 μm (B′), 10 μm (C,D). Error bars in E represent s.d.

To further validate that our scale explant is an accurate model for observing cutaneous axon degeneration and engulfment, we asked whether *mpeg1*^+^ cells engulfed axonal debris *in vivo.* To test this, we used two cutaneous injury models in adult fish combined with previous methods of intubation and imaging to observe *mpeg1*^+^ cells in living animals (Fig. 3A). First, we snipped individual scales with micro scissors, which created widespread damage in the epidermis and ultimately caused WD. Axon degeneration was observed 2-4 h after injury, consistent with the timing of WD *ex vivo* (Fig. 3B; Movies 5 and 6)*.* Following axon degeneration, we observed *mpeg1*^+^ cells engulfing axonal debris (Fig. 3B′, insets; Movies 5 and 6), providing evidence that *mpeg1^+^* cells clear cutaneous axonal debris *in vivo.* Second, we used precise laser axotomy to sever individual scale nerves and trigger WD (Fig. 3C). This more targeted approach allowed us to predict which axons would undergo WD, allowing us to quantify the relative contributions of keratinocytes and *mpeg1^+^* cells to debris engulfment *in vivo*. These data closely matched results from the scale explant assays (Fig. 3D,E). Notably, within these imaging periods, we did not observe recruitment of exogenous *mpeg1^+^* cells to sites of injury or WD. Together, these data strongly suggest that resident *mpeg1^+^* Langerhans cells and/or metaphocytes internalize axon debris following WD in adult epidermis.

### Langerhans cells constitute the *mpeg1*^+^ cell population that engulfs axon debris

As described above, *mpeg1* reporters label multiple cell types in the adult epidermis. This includes both Langerhans cells, skin-resident immune cells known mainly for their antigen-presenting properties in mammals (Doebel et al., 2017; Kaplan, 2017), and metaphocytes, a recently identified epidermal cell type in zebrafish (Alemany et al., 2018; Lin et al., 2019). A subset of dendritic cells isolated from adult zebrafish skin contains Birbeck granules (Lugo-Villarino et al., 2010), a defining characteristic of Langerhans cells (Birbeck et al., 1961; Valladeau et al., 2000), suggesting that zebrafish skin contains bona fide Langerhans cells. Interestingly, a previous report suggested that metaphocytes uptake soluble antigens, which they transfer to Langerhans cells via an apoptosis–phagocytosis mechanism (Lin et al., 2019).

**Fig. 4.**
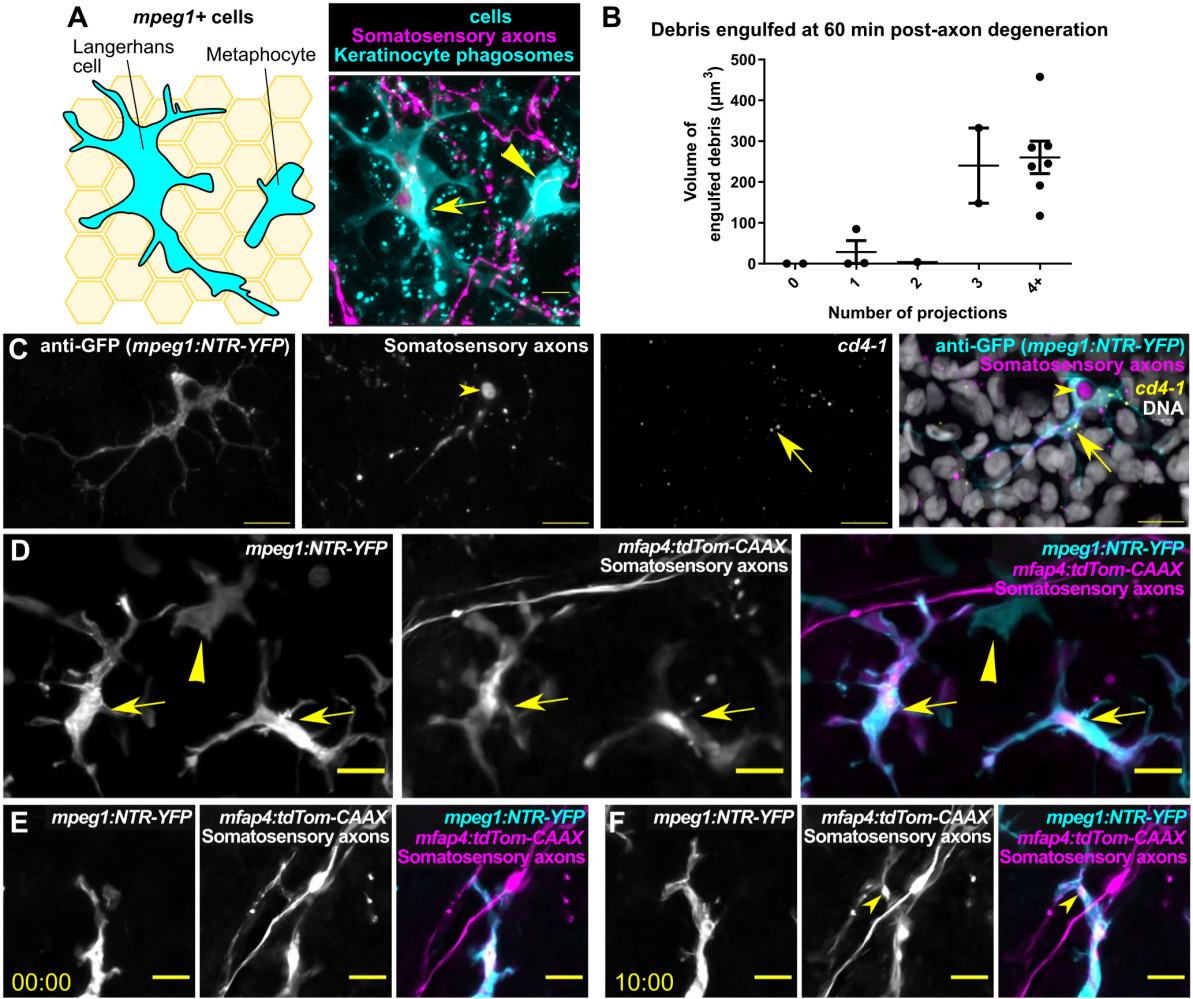
**Langerhans cells represent the *mpeg1^+^* cell type that engulfs cutaneous axon debris.** (A) Schematic (left) and corresponding fluorescent image (right) comparing the morphology of a Langerhans cell (arrow) and a metaphocyte (arrowhead). (B) Quantification of axonal debris engulfed relative to number of protrusions in *mpeg1^+^* cells 60 min post-scale pluck-induced axon degeneration, *n*=12 scales from *N*=9 fish. (C) Fluorescence *in situ* hybridization with HCR probes against *cd4-1* following scale pluck-induced axon degeneration. Arrows indicate *cd4-1* expression in *mpeg1^+^* cell; arrowheads indicate engulfed axonal debris*.* (D) Fluorescence images of the scale epidermis in an adult expressing *Tg(mpeg1:NTR-EYFP)*, *Tg(mfap4:tdTom-CAAX)* and a somatosensory axon reporter [*Tg(p2rx3a:mCherry)*]. Arrows indicate *mpeg1^+^/mfap4^+^* cells (Langerhans cells); arrowheads indicate an *mpeg1^+^* only cell (metaphocyte). (E,F) Still images of the scale epidermis in a *Tg(mpeg1:NTR-EYFP);Tg(mfap4:tdTom-CAAX);Tg(p2rx3a:mCherry)* adult before and during scale pluck-induced axon degeneration. Arrowheads indicate engulfed axonal debris. Time denotes mm:ss. Scale bars: 5 μm (A), 10 μm (C-F). Error bars in B denote s.d.

To distinguish between possible contributions of Langerhans cells and metaphocytes to axonal debris engulfment, we took three parallel approaches. First, we used a previously described methodology based on morphological differences (Kuil et al., 2020). In this approach, Langerhans cells are distinguished by multiple, branched protrusions, whereas metaphocytes have fewer, less complex protrusions (Fig. 4A). We quantified the volume of internalized debris within individual *mpeg1^+^ cells* at 60 min post-axon degeneration and found that debris engulfment positively correlated with protrusion number (Fig. 4B), suggesting that the subset of *mpeg1^+^* cells we observed engulfing axonal debris was Langerhans cells. Second, we performed fluorescent *in situ* hybridization for *cd4-1*, a gene expressed by Langerhans cells but not metaphocytes (Lin et al., 2019). Scales were removed and fixed 4 h post-removal, a time point at which axons are degenerating and Langerhans cells contain axon debris. *cd4-1* signal colocalized with *mpeg1^+^* cells that had internalized axon debris (Fig. 4C). Third, we used a transgenic approach to differentially label Langerhans cells and metaphocytes during axon degeneration and debris engulfment. RNA-sequencing transcriptional profiling indicates that Langerhans cells, but not metaphocytes, express the gene *mfap4* (also known as *mfap4.1*) (Kuil et al., 2020; Lin et al., 2019). To determine whether a previously generated *mfap4* transgene could be used to label Langerhans cells, we crossed *Tg(mfap4:tdTomato-CAAX)* (Walton et al., 2015) fish with *Tg(mpeg1:NTR-EYFP);Tg(p2rx3a:mCherry)* fish. In these triple transgenic animals, we expected that Langerhans cells would be *mpeg1^+^/mfap4^+^*, whereas metaphocytes would be *mpeg1^+^/mfap4^−^*. Indeed, we found that *mfap4^+^* cells that were also *mpeg1^+^* had the highly branched morphology consistent with Langerhans cells, whereas metaphocytes were only *mpeg1^+^* (Fig. 4D). Removing scales and imaging for axonal debris engulfment revealed that *mpeg1^+^/mfap4^+^* cells specifically engulfed axonal debris (Fig. 4E,F). Based on these results, we concluded that Langerhans cells are the primary adult cell type responsible for engulfing cutaneous axon debris.

### Keratinocytes do not compensate for the loss of Langerhans cells

**Fig. 5.**
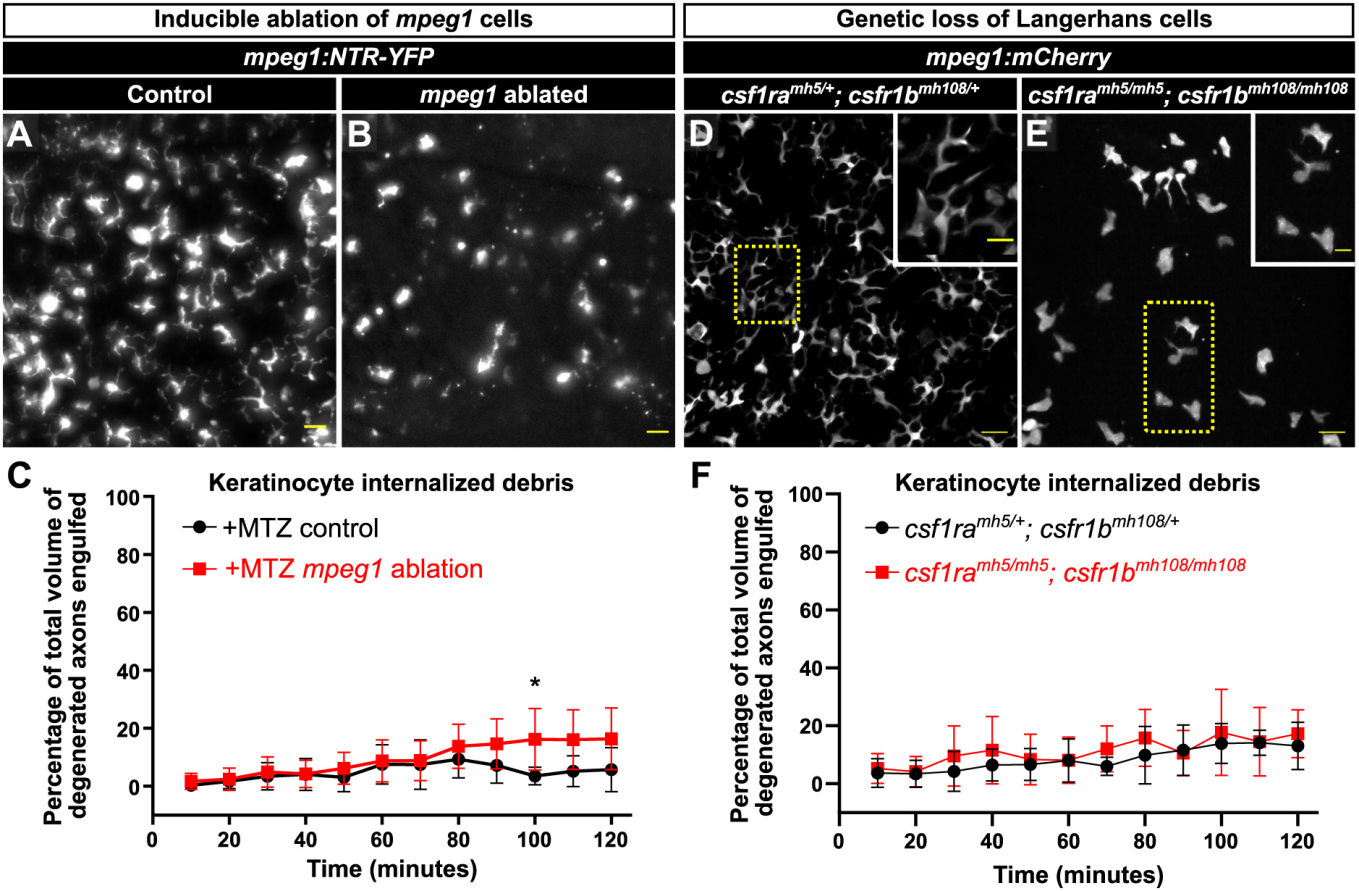
**Keratinocytes do not compensate for the absence of Langerhans cells.** (A,B) Representative widefield images of the scale epidermis in *Tg(mpeg1:NTR-EYFP)* adults after 3 days of mock treatment (A) or 7 mM MTZ treatment to ablate *mpeg1^+^* cells (B). *mpeg1^+^* cells: 255.1±133.17 and 73.8±23.28 (mean±s.d.) after 3 days of mock or MTZ treatment, respectively. (C) Quantification of debris engulfed by keratinocytes after 3 days of exposure to MTZ in animals with or without *Tg(mpeg1:NTR-EYFP)* (denoted as +MTZ *mpeg1* ablation or +MTZ control, respectively). Two-way ANOVA followed by Bonferroni tests determined significance of differences between (+MTZ−NTR) and (+MTZ+NTR) conditions. *P<0.05. *n*=4-10 ROIs for +MTZ−NTR (from three fish, six scales) and *n*=15-16 ROIs for +MTZ+NTR (from seven fish, 13 scales). (D,E) Representative confocal images of *mpeg1^+^* cells in the scale epidermis from adults of the indicated genotypes. Note the lack of *mpeg1^+^* cells with the dendritic morphology of Langerhans cells in the *csf1ra^mh5/mh5^; csf1rb^mh108/mh108^* mutant epidermis. Yellow dotted line boxes denote the regions magnified in insets. (F) Quantification of debris engulfed by keratinocytes in adults of the indicated genotypes. *n*=6-11 ROIs for *csf1ra^mh5/+^; csf1rb^mh108/+^* (from three fish, four scales) and *n*=10 ROIs for *csf1ra^mh5/mh5^; csf1rb^mh108/mh108^* (from three fish, five scales). Scale bars: 20 μm (A,B,D,E), 10 μm (D,E, insets). Error bars in C and F represent s.d.

In direct contrast to larval skin, where keratinocytes are the primary phagocytes for axon debris (Han et al., 2014; Rasmussen et al., 2015), we found that adult keratinocytes largely do not contribute to debris clearance (Figs 2 and 3). Does the presence of highly phagocytic Langerhans cells in adult skin inhibit the ability of keratinocytes to engulf axon debris? To address this question, we used a previously described transgenic system [*Tg(mpeg1:NTR-EYFP)* (Petrie et al., 2014)] to conditionally ablate *mpeg1^+^* cells, comprising both Langerhans cells and metaphocytes. *Tg(mpeg1:NTR-EYFP)* fish express the nitroreductase (NTR) enzyme fused to EYFP under control of the *mpeg1* promoter. Upon exposure to the prodrug metronidazole (MTZ), NTR converts MTZ into a toxic compound, thereby killing *mpeg1^+^* cells. We found that treating *Tg(mpeg1:NTR-EYFP)* adults with 7 mM MTZ for 3 days ablated most *mpeg1^+^* cells (Fig. 5A,B). As controls, we treated siblings without *Tg(mpeg1:NTR-EYFP)* with MTZ. By removing scales and imaging axon degeneration following MTZ exposure, we observed only a small increase in axon engulfment by keratinocytes in the absence of *mpeg1^+^* cells (Fig. 5C).

As a parallel strategy, we next examined mutants that lack developmental colonization of the epidermis by Langerhans cells, a process that requires Colony stimulating factor-1 receptor (Csf1r) (Dai et al., 2002; Kuil et al., 2020). We first confirmed that animals with loss-of-function mutations in both of the zebrafish Csf1r paralogs [*csf1ra^mh5/mh5^; csf1rb^mh108/mh108^* (Caetano-Lopes et al., 2020)] lacked Langerhans cells (Fig. 5D,E). Using *csf1ra^mh5/mh5^; csf1rb^mh108/mh108^* or *csf1ra^mh5/+^; csf1rb^mh108/+^* animals, we next repeated the engulfment assays and found that genetic ablation of Langerhans cells resulted in no change in the amount of debris engulfed by keratinocytes (Fig. 5F). We conclude that keratinocytes do not engulf more axon debris in the absence of Langerhans cells.

## DISCUSSION

In this study, we combine cutaneous injuries in adult zebrafish with high-resolution confocal imaging to examine the mechanisms of axonal debris clearance following WD. We establish *in vivo* and *ex vivo* techniques for analyzing axonal debris engulfment with subcellular resolution in adult skin. In contrast to studies in larval models, we show that keratinocytes do not play a major role in axonal debris removal in adult epidermis. Instead, we demonstrate a new macrophage-like role for Langerhans cells in the clearance of axonal debris following several types of injury-induced WD. We observed that Langerhans cells do not prematurely attack axons, but rather engulf debris only after an axon has undergone WD. Prior to our study, it had not been fully understood how axon repair begins following WD in the epidermis. We now propose that, as skin-resident macrophages, Langerhans cells are the first responders to axon injury and begin the repair process by clearing axonal debris. Combined, our data suggest differences in phagocytic properties in larval versus adult skin and reveal an unappreciated role for Langerhans cells in promoting skin homeostasis after injury.

### Developmental decline in keratinocyte phagocytosis

We were surprised to find that adult keratinocytes had a greatly reduced phagocytic capability compared to their larval counterparts (Fig. 2A; Fig. S1A). Several possible explanations exist for this apparent decline. First, the bilayered epidermis of larval zebrafish is much simpler than the stratified adult epidermis. A more constrained three-dimensional and/or adhesive environment could decrease the ability for keratinocytes to rearrange their plasma membranes in order to engulf debris. Meanwhile, Langerhans cells possess dynamic cellular protrusions (Nishibu et al., 2006), allowing them to navigate this complex environment to seek out and engulf axonal debris. A second possibility is that keratinocytes lose phagocytic competency through downregulation of the necessary machinery as organogenesis progresses. Controlled studies comparing larval and adult skin should be performed to fully investigate this scenario. Third, infiltration of immune cells, such as Langerhans cells, specialized for debris removal could trigger a change in keratinocyte gene expression. A study examining the transcriptomes of keratinocytes in the presence or absence of Langerhans cells revealed differential gene expression in keratinocytes (Su et al., 2020). However, our analysis of these results showed no upregulation of pro-engulfment genes in the absence of Langerhans cells, consistent with our observations that the absence of Langerhans cells does not promote keratinocyte phagocytosis (Fig. 5).

### Langerhans cells and tissue-resident macrophage-like functions

Historically, Langerhans cells have been studied for their role as dendritic cells with antigen-presenting capabilities. In this context, Langerhans cells emigrate from the epidermis to lymph nodes, where they instruct adaptive immune responses (Doebel et al., 2017; Kaplan, 2017). However, roles for Langerhans cells *in situ* within the epidermis remain largely enigmatic (West and Bennett, 2017). Recent works have revealed that Langerhans cells share developmental origins with tissue macrophages (He et al., 2018; Sheng et al., 2015). Despite these recent advances in understanding the origins of Langerhans cells, their functional similarities to macrophages remain largely unknown. Examples of Langerhans cells performing phagocytic functions traditionally associated with macrophages, such as engulfment of apoptotic corpses, are currently limited to a handful of examples (Bauer et al., 2012; Hatakeyama et al., 2017; Lin et al., 2019), and their role in wound healing has only recently begun to be investigated (Wasko et al., 2022). Our work unambiguously establishes a new macrophage-like role for Langerhans cells in maintaining skin homeostasis by using their long protrusions to engulf debris from degenerating axons. Future work will address the mechanisms underlying protrusion motility and the phagocytic pathways used by Langerhans cells during debris engulfment, including the machinery involved in recognizing, engulfing and degrading axonal debris. Although not the focus of this study, an interesting future area of exploration will be to examine whether Langerhans cells communicate with T cells to present antigen following WD, or whether Langerhans cells process and degrade axon debris without involvement of other cells.

A growing body of literature argues that Langerhans cells and microglia, tissue-resident macrophages of the central nervous system, share a number of characteristics. Langerhans cells and microglia both require interleukin-34 (Il-34) and Csf1r signaling for tissue infiltration (Kuil et al., 2019, 2020; Wang et al., 2012; Wu et al., 2018). Both cell types have dynamic cellular protrusions that surveil the environment, and transcriptomic studies indicate that they have overlapping gene expression profiles (Mass et al., 2016). Intriguingly, our work shows that Langerhans cells are phagocytes of neuronal debris, similar to microglia in multiple vertebrate systems. Microglia have been well characterized for their roles in synapse remodeling during development, where they can prune developing arbors according to neuronal activity (Bachiller et al., 2018). We hypothesize that Langerhans cells likely play similar roles in shaping cutaneous axon arbors during skin organogenesis.

We used two complementary approaches to examine keratinocyte phagocytosis in the absence of Langerhans cells. Each of our approaches has potential caveats. A limitation of the *mpeg1^+^* ablation strategy is that it reduces both metaphocyte and Langerhans cell numbers. Metaphocytes may influence debris engulfment by either directly or indirectly affecting Langerhans cells or keratinocytes. Ablation of metaphocytes using a metaphocyte-specific transgene (Lin et al., 2020) could address this possibility. If metaphocytes are involved in the response to WD, their ablation would see decreases in axonal debris internalization by Langerhans cells, keratinocytes or potentially both. In parallel, we compared keratinocyte responses to WD in *csf1ra^mh5/mh5^; csf1rb^mh108/mh108^* mutants and *csf1ra^mh5/+^; csf1rb^mh108/+^* controls. It is worth noting that certain *csf1r* mutant alleles have dominant-negative effects on microglial numbers (Berdowski et al., 2022). Although no dominant-negative phenotypes of the alleles we used have previously been reported (Caetano-Lopes et al., 2020), we cannot rule out this possibility. Future work better characterizing these mutant alleles and reproducing our own work in additional genetic backgrounds will aid in characterizing roles for Langerhans cells and metaphocytes in adult zebrafish.

### Strengths and limitations of cutaneous injury models

We note that the *in vivo* and *ex vivo* approaches for tracking cutaneous WD established here have relative strengths and limitations. Strengths of the *in vivo* approaches include the ability to track contributions of exogenous cell types to injury responses and the potential to assess axon reinnervation, neither of which are possible in the explant system. The micro scissors approach is a simple, low-cost method to injure scales and epidermis that has previously been used to monitor bone healing (Kobayashi-Sun et al., 2020). However, damage cannot be targeted to specific cells, making it difficult to predict which axons will undergo WD, thereby effectively limiting imaging resolution. By contrast, laser axotomy can sever single nerves or axons, but relies on specialized equipment not commonly available. Monitoring WD *in vivo* requires anesthesia and intubation for imaging, which may have unknown effects on the injury response. In contrast to the *in vivo* approaches, the scale explant model reliably severs all cutaneous nerves and allows for the highest spatiotemporal imaging resolution. The scale explant system is also readily amenable to small-molecule screens to identify new compounds or pathways that influence WD or debris engulfment.

### Summary

In summary, we show that Langerhans cells clear axonal debris following WD in the epidermis. Of note, Langerhans cell ablation has been associated with a decrease in cutaneous axon density in mouse (Doss and Smith, 2014; Zhang et al., 2021), suggesting that skin innervation or axon maintenance requires Langerhans cells. In addition, Langerhans cells may have roles in mediating or exacerbating peripheral neuropathies, conditions in which epidermal innervation is decreased, resulting in perturbations to skin sensation. Peripheral neuropathies, such as chemotherapy-induced neuropathy and diabetic peripheral neuropathy, result in altered numbers of Langerhans cells in the epidermis (Siau et al., 2006; Stojadinovic et al., 2013). The relationship between Langerhans cells and polyneuropathies is currently poorly understood, but is of potential clinical significance. It is possible that the increase in Langerhans cells in these conditions is due to ongoing nerve damage, but the prolonged or persistent presence of Langerhans cells may exacerbate inflammation and hinder axon regeneration. Zebrafish models of chemotherapy-induced and diabetic neuropathy exist (Cirrincione et al., 2020; Kimmel et al., 2015), but how Langerhans cell numbers or activity change in response to neuropathy in these models has not been studied. Our zebrafish injury models could have relevance for identifying mechanisms that mediate the initiation or progression of peripheral neuropathies, and future studies in zebrafish could provide a unique perspective on the role of Langerhans cells in these human diseases.

## MATERIALS AND METHODS

### Zebrafish and husbandry

Zebrafish were housed at 26-27°C on a 14/10 h light cycle. The strains used are listed in Table S1. Animals aged 6-18 months of either sex were used in this study. All zebrafish experiments were approved by the Institutional Animal Care and Use Committee at the University of Washington (Protocol #4439-01).

### Genotyping

Adult fish were genotyped to ensure that both *Tg(krt4:EGFP-2xFYVE)* and *Tg(ΔNp63:EGFP-2xFYVE)* were present. The primer pairs used were *krt4seq-fwd/gfp-sR* and *p63-atg-1kb-fwd/gfp-sR* to detect *Tg(krt4:EGFP-2xFYVE)* and *Tg(ΔNp63:EGFP-2xFYVE)*, respectively. The *csf1ra^mh5^* and *csf1rb^mh108^* alleles were genotyped with high-resolution melt analysis using the primer pairs *csf1ra-72-fwd/csf1ra-72-rev* and *csf1rb-100-fwd/csf1rb-100-rev*, respectively. Primer sequences can be found in Table S1.

### Transgene construction

The *ΔNp63:EGFP-2xFYVE* BAC was created by modifying the previously generated BAC DKEY-263P13-iTol2-amp (Rasmussen et al., 2015). The predicted *ΔNp63* start codon was replaced by a *EGFP-2xFYVE-pA-KanR* cassette using a previously described protocol (Suster et al., 2011). The *pDEST-krt4:EGFP-2xFYVE-pA* plasmid was assembled using Gateway recombination of *p5E-krt4* (O'Brien et al., 2012), *pME-EGFP-2xFYVE* (Rasmussen et al., 2015), *p3E-polyA* and *pDestTol2pA2* (Kwan et al., 2007). *Tg(krt4:EGFP-2xFYVE)^w265Tg^* and *TgBAC(ΔNp63:EGFP-2xFYVE)^w266Tg^* were created by injecting *tol2* mRNA, which was transcribed from pCS2-zT2TP (Suster et al., 2011), and either plasmid or BAC DNA into one-cell stage embryos and screening adults for germline transmission.

### Scale removal

For scale removal, adult fish were anesthetized in system water containing 200 µg/ml buffered tricaine, and forceps were used to remove individual scales. Following scale removal, animals were recovered in system water.

### Microscopy and live imaging

An upright Nikon Ni-E A1R MP+ confocal microscope was used for all experiments. A 25× water dipping objective (1.1 NA) was routinely used. Unless otherwise stated, scales were removed and placed onto dry 6 mm plastic dishes, epidermis side up, and allowed to adhere for 1 min before adding L-15 medium pre-warmed to room temperature. Scales were incubated at 26°C for 90 min followed by imaging, which was performed at room temperature (23°C).

### Larval *in vivo* axotomy

Larval (5 days post-fertilization) fish were anesthetized in system water containing 80 µg/ml tricaine. Fish were mounted laterally in 1% low-melt agarose dissolved in system water. Trigeminal axons were located and severed using a UGA-42 Caliburn pulsed 532 nm laser (Rapp OptoElectronic). The laser was focused through a 25× objective at 4× zoom. Ablation was produced in the focal plane using 50% power in a circular region of interest (ROI) drawn the diameter of an axon, illuminating eight random points within the circle for 1 s each using a custom NIS-Elements macro. This was repeated once, for a total of two firings on each axon. Axons were examined 5 min post-firing to confirm severing was achieved.

### Adult *in vivo* cutaneous injury and imaging

For *in vivo* micro scissor injury and imaging, adult fish were anesthetized in system water containing 200 µg/ml tricaine. Sterile micro scissors were used to cut through a scale on the lateral trunk. Fish were subsequently immobilized and mounted in a custom imaging chamber using 1% agarose dissolved in system water as described (Xu et al., 2015). Fish were intubated with aerated system water containing 120 µg/ml buffered tricaine. The chamber was placed under a 16× water dipping objective (0.8 NA), and the site of injury was located. Imaging was performed using an environmental enclosure (OKO). The water temperature within the imaging chamber was 23°C, as measured using an immersible digital probe. After the imaging session, fish were recovered with aerated system water.

For *in vivo* axotomy, fish were mounted into the imaging chamber and microscopy enclosure as described above. Target axons were located and ablated using a UGA-42 Caliburn pulsed 532 nm laser (Rapp OptoElectronic). The laser was focused through a 16× objective at 4× zoom. Ablation was produced in the focal plane using 75% power in a circular ROI drawn the diameter of an axon, illuminating eight random points within the circle for 2 s each using a custom NIS-Elements macro. This was repeated twice, for a total of three firings on each axon. Axons were examined 5 min post-firing to confirm that severing was achieved.

### Chemical treatments and live-cell staining

For FK866 and DSRM-3716 treatments, scales were removed and immediately placed in L-15 medium containing 10 µM FK866 or 10 µM DSRM-3716. Scales were incubated for 90 min at 26°C before imaging commenced.

For MTZ treatments, fish were placed in system water containing 7 mM MTZ for 3 days. Fish were housed at a density of one fish per liter. MTZ solution was replaced daily. Fluorescent *z*-stacks of individual scales were acquired on an Axio Zoom.V16 (Zeiss), and the number of *mpeg1^+^* cells was quantified after 3 days of mock or MTZ treatment to confirm transgene-mediated ablation.

For LysoTracker staining, scales were removed and placed in a 1.5 ml tube containing 2 nM LysoTracker Deep Red/L-15 medium. Scales were incubated in the dark for 30 min at 26°C, washed quickly in L-15 medium and placed onto a 6 mm dish, epidermis side up. Scales were incubated in L-15 medium at 26°C in the dark for a further 60 min before imaging commenced.

### Image analysis

To quantify percentage axon degeneration in Fig. 1, random ROIs were selected and tracked every 15 min. The total axon number was used to calculate the percentage of axons undergoing degeneration. Degeneration index was calculated as previously described (Sasaki et al., 2009). Briefly, images were thresholded in ImageJ, and total axon intensity and degenerated axon intensity were calculated. The degeneration index represents the fraction of degenerated axon intensity over the total axon intensity.

To quantify debris engulfment, individual ROIs containing Langerhans cells were identified, and the Imaris Surfaces function was used. Surfaces were created for Langerhans cells, keratinocyte EGFP-2xFYVE^+^ phagosomes, and axons. Additional Surfaces were created using the same intensity threshold as the axon Surface, then filtered to only include material inside the ‘Langerhans cell’ Surface or ‘FYVE’ Surface. Images were manually inspected and corrected to ensure that no erroneous material was counted as inside a Surface. Volumes for total axon volume and engulfed volume were recorded every 10 min, and percentage debris engulfed was calculated.

To differentiate metaphocytes from Langerhans cells in Fig. 3, the numbers of cellular protrusions on *mpeg1^+^* cells were counted at 60 min post-axon degeneration. A protrusion was defined as a process that extended ≥5 μm from the cell body.

### Hybridization chain reaction (HCR)

A custom *cd4-1* probe set (set size, 20; amplifier, B3) was designed using the insitu_probe_generator software (Kuehn et al., 2022) and accession number XM_005173496.4. For HCR on adult zebrafish scales, minor alterations were made to a previously described protocol (Ibarra-García-Padilla et al., 2021). Briefly, scales from *Tg(mpeg1:NTR-EYFP);Tg(p2rx3a:mCherry)* adults were plucked and incubated in L-15 medium at 28°C for 4 h to ensure axon degeneration. Scales were fixed in 4% paraformaldehyde (PFA) overnight at 4°C, with 5-10 scales per 1.5 ml tube. Scales were washed 3× in 1× PBS and then dehydrated and permeabilized with 2×10 min washes in 100% methanol. The samples were stored at −20°C overnight. To rehydrate the samples, a series of graded methanol/PBS 0.1% Tween-20 (PBST) washes were used for 5 min each: 75% methanol:25% 1× PBST, 50% methanol:50% 1× PBST, 25% methanol:75% 1× PBST and, finally, 2× washes in 100% 1× PBST. To further permeabilize the scales, samples were incubated in 10 μg/ml proteinase K diluted in 1× PBST for 10 min. Samples were washed 3× in 1× PBST without incubation, and then post-fixed with 4% PFA for 20 min. After post-fixation, samples underwent 5×5 min washes with 1× PBST. Samples were then pre-hybridized with Molecular Instruments HCR hybridization buffer at 37°C for 30 min. After pre-hybridization, samples were incubated with 2 pmol of the probe set diluted in hybridization buffer for 16 h at 37°C. To remove the probe mixture solution, samples were washed 4× for 15 min each with probe wash buffer at 37°C. Samples were washed 2× for 5 min with 5× SSC+0.1% Tween-20 and then treated with probe amplification buffer for 30 min at room temperature. Samples were washed into hairpin amplification buffer containing snap cooled amplifier hairpins and were incubated at room temperature, protected from light, overnight. Samples were then washed with successive 5× SSC+0.1% Tween-20 washes: 2× washes for 5 min, 2× washes for 30 min and 1× wash for 5 min. Finally, samples underwent 3×5 min washes with 1× PBST. Anti-GFP staining was performed by first blocking the scales in a 5% normal goat serum (NGS) blocking solution for 2 h. After 2 h, the blocking solution was replaced with a 1:500 dilution of anti-GFP antibody diluted in NGS, and samples were incubated at 4°C overnight. Then, the scales were washed 6× for 30 min in PBST prior to incubating the scales in 1:1000 anti-rabbit secondary antibody, also diluted in NGS, overnight at 4°C. Finally, the scales were washed 6× for 30 min in PBST and then stained with 1× DAPI for 10 min prior to mounting for confocal imaging.

### Statistical analysis

GraphPad Prism was used to generate graphs and perform statistical analyses. Tests used and number of animals, scales or cells/ROIs are described in each figure legend and in Table S2.

### Key resources

Key resources are listed in Table S1.

## Supplementary Material

10.1242/dmm.049911_sup1 Supplementary information
