# Supplementary material for: Zebrafish cutaneous injury models reveal that Langerhans cells engulf axonal debris in adult epidermis
Source: Dis Model Mech. 2023 Apr 3;16(4):dmm049911. doi: 10.1242/dmm.049911 (PMC10110399; doi:10.1242/dmm.049911)
Supplement: Supplementary information [file dmm-16-049911-s1.pdf]

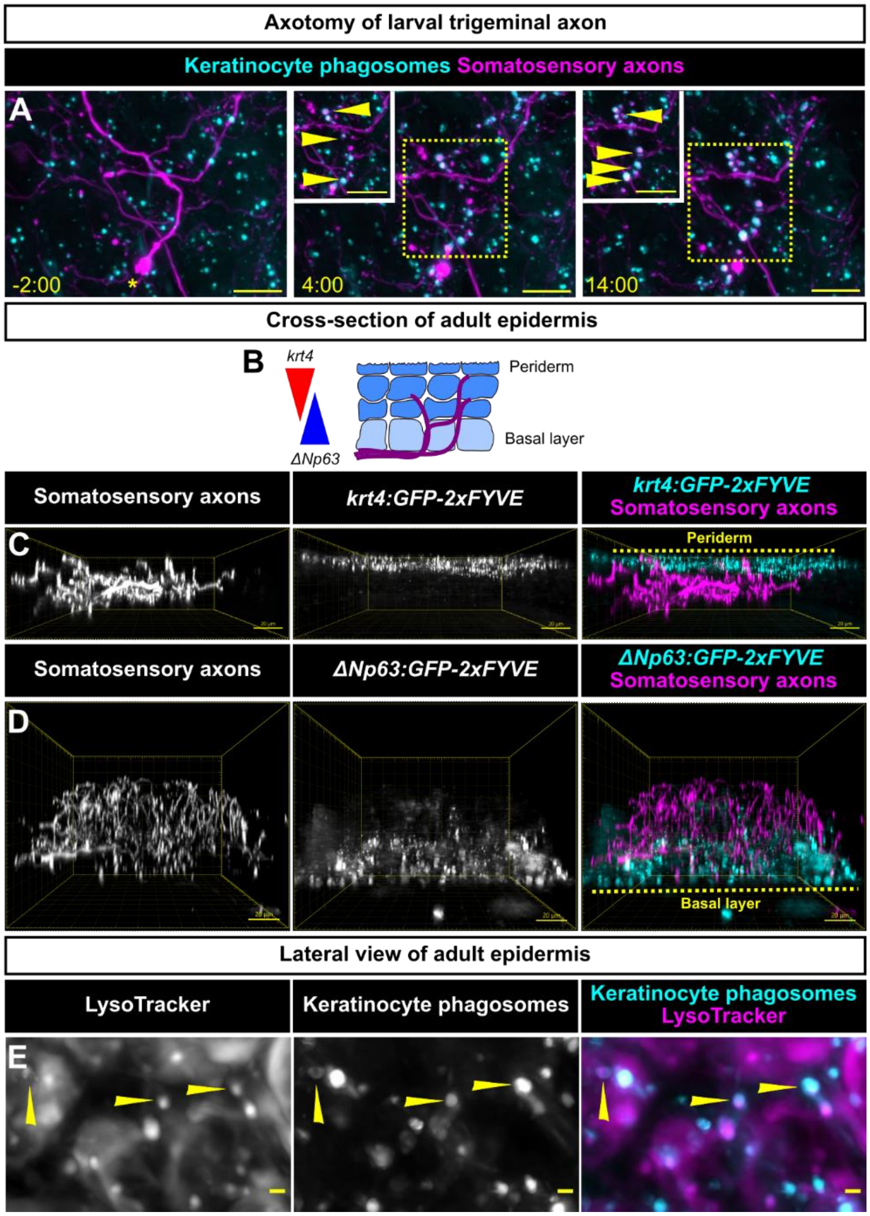

Fig. S1. Characterization of epidermal keratinocyte transgenes. (A) Validation of keratinocyte phagosome transgenes in larval zebrafish. Laser axotomy of axons emanating from trigeminal neurons results in WD, followed by axonal debris internalization into keratinocyte phagosomes. Asterisk denotes site of axotomy. Shown are representative confocal micrographs before and during axon degeneration. Time mm:ss. (B) Schematic depicting gradients of transgene expression patterns in adult epidermis. (C, D) Reconstructed cross-sections showing expression of *Tg(krt4:EGFP-2xFYVE)* and *TgBAC(ΔNp63:EGFP-2xFYVE)* in adult epidermis. (E) LysoTracker co-localization with EGFP-2xFYVE+ structures (arrowheads). Scale bars: 10 μm (A, C,D), 1 μm (E).

Disease Models & Mechanisms • Supplementary information

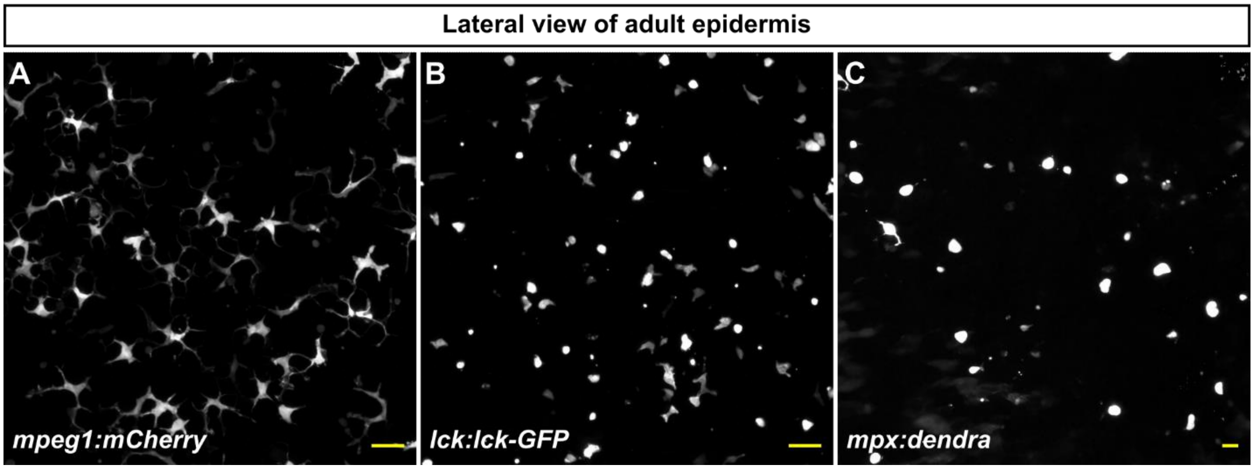

Fig. S2. Identification of different immune cell populations present in adult epidermis. (A-C) Lateral confocal views of the adult *ex vivo* scale epidermis expressing the indicated transgenes. Scale bars: 20 μm.

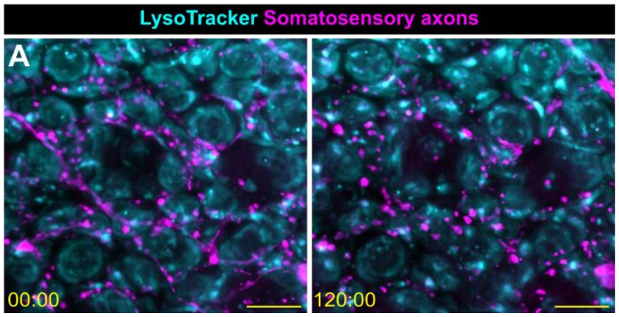

Fig. S3. Axonal debris does not compartmentalize into other Lysotracker+ vesicles after 2 hours. Lysotracker staining did not reveal other potential cell populations engulfing debris, and debris remains unengulfed after 2 hours. Time stamp mm:ss, scale bar 10 μm.

Table S1. Key resources

Click here to download Table S1

Table S2. Replicate details

Click here to download Table S2

Disease Models & Mechanisms • Supplementary information

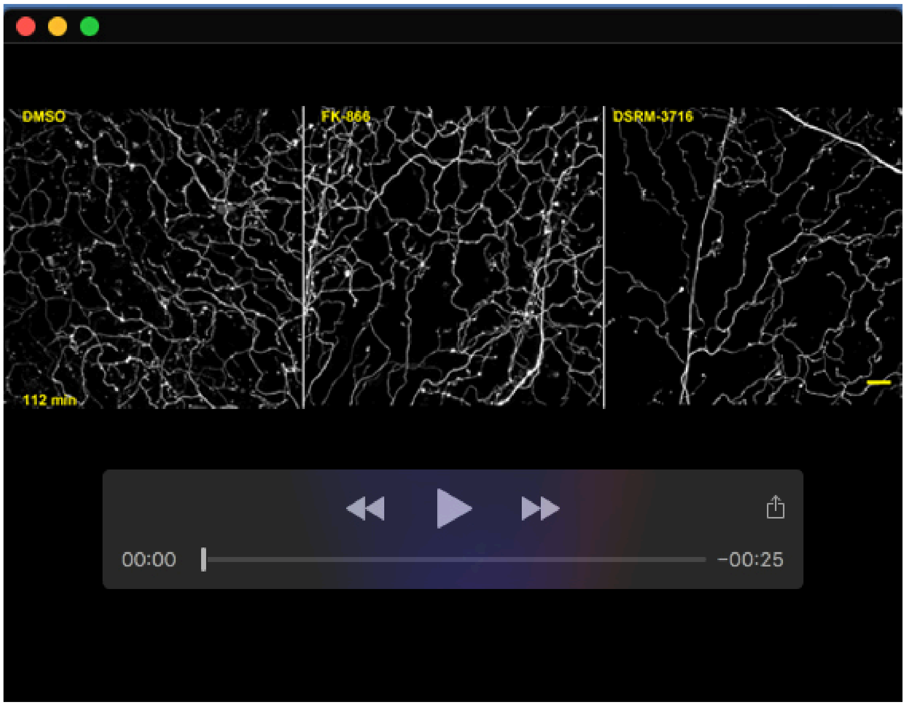

Movie 1. Time-lapse microscopy of *Tg(p2rx3a:mCherry)*+ axons in control conditions or treated with FK866 or DSRM-3716.

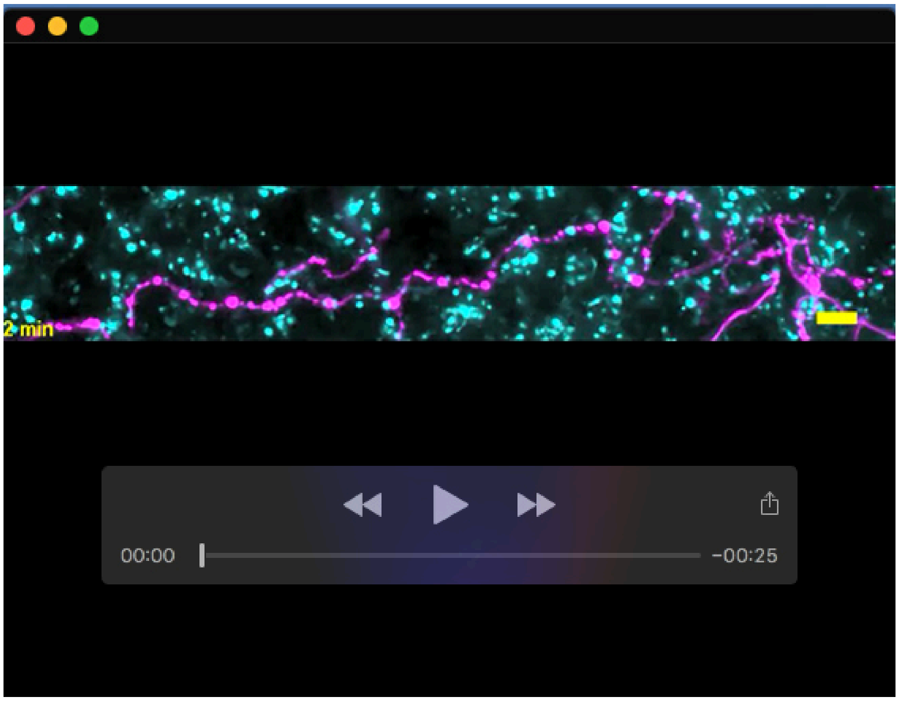

Movie 2. Time-lapse microscopy of *Tg(krt4:EGFP-2xFYVE);TgBAC(ΔNp63:EGFP-2xFYVE)*+ phagosomes engulfing a small quantity of *Tg(p2rx3a:mCherry)*+ axon debris. Time 0 denotes onset of axon degeneration.

Disease Models & Mechanisms • Supplementary information

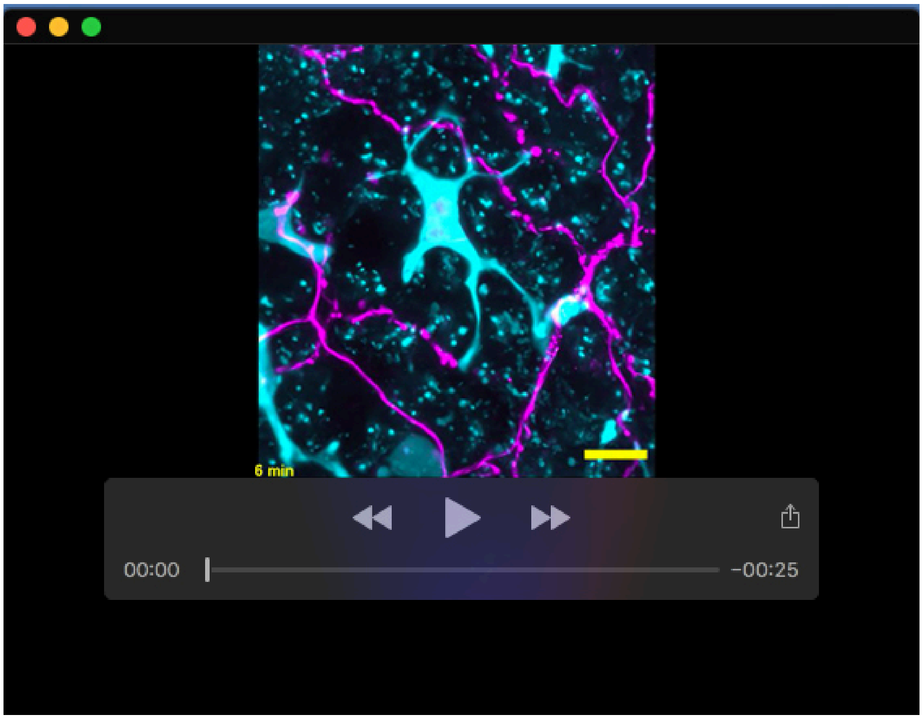

Movie 3. Time-lapse microscopy of *Tg(p2rx3a:mCherry)*+ debris being internalized by *Tg(mpeg1:NTR-EYFP)* cells. Time 0 denotes onset of axon degeneration.

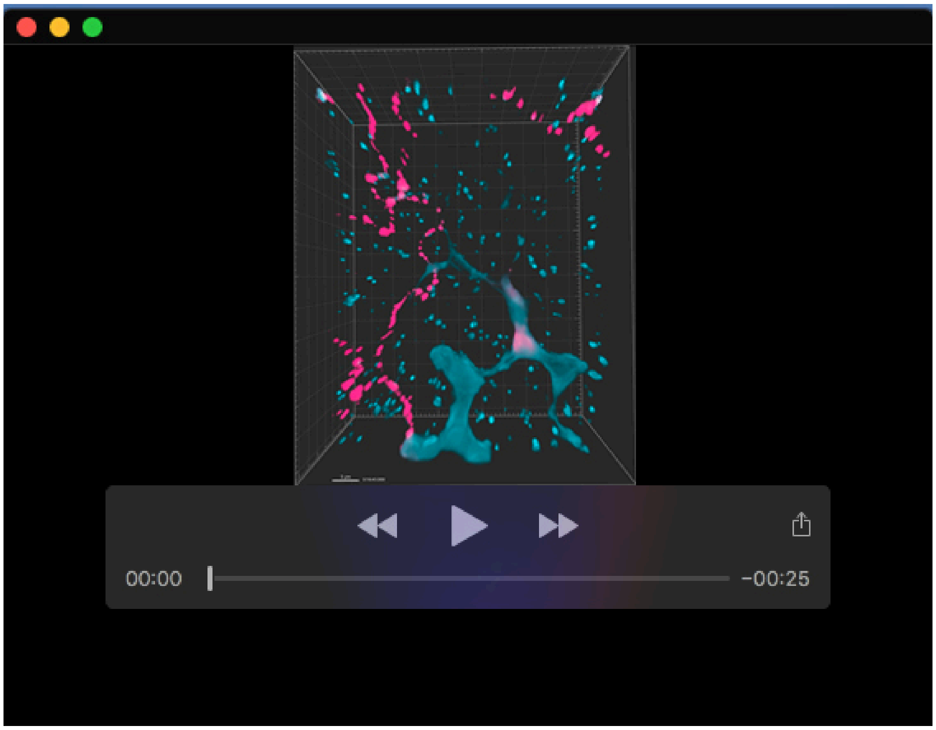

Movie 4. Imaris volume-blended time-lapse microscopy of *Tg(p2rx3a:mCherry)*+ debris being internalized by *Tg(mpeg1:NTR-EYFP)* cells.

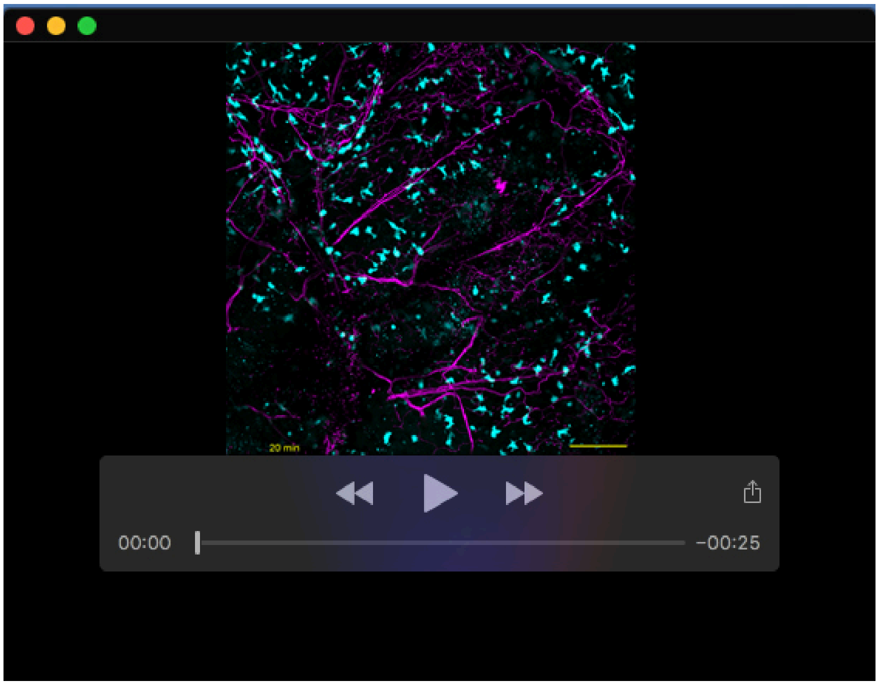

Movie 5. *In vivo* time-lapse microscopy of a scale injury in *Tg(p2rx3a:mCherry;Tg(mpeg1: NTR-EYFP)* animals.

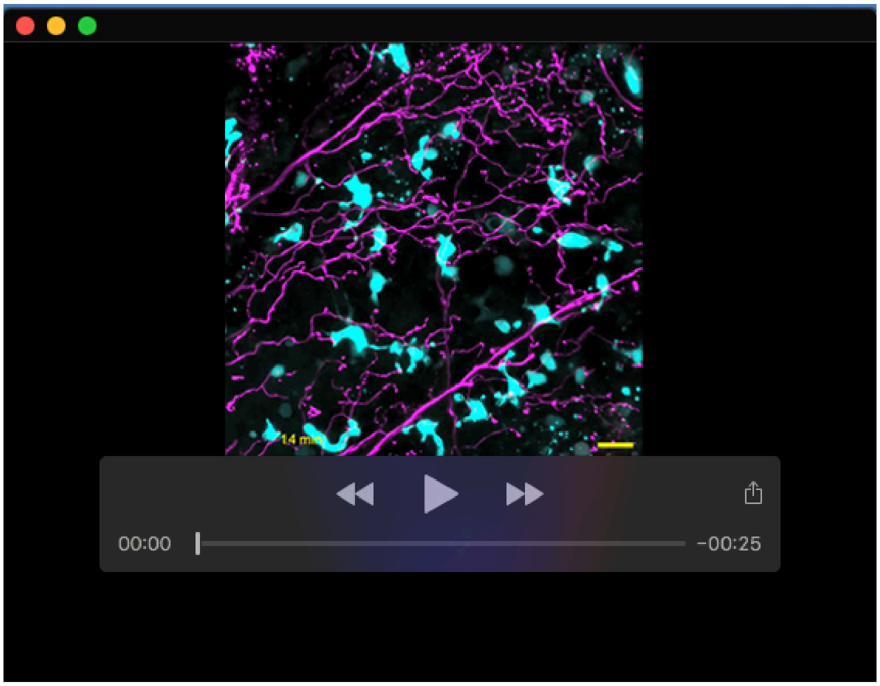

Movie 6. Cropped and magnified view from video 5 showing *Tg(p2rx3a:mCherry)+* debris being internalized by *Tg(mpeg1:NTR-EYFP)* cells.

Disease Models & Mechanisms • Supplementary information

Supplementary References

Ellett, F., Pase, L., Hayman, J. W., Andrianopoulos, A. and Lieschke, G. J. (2011). mpeg1 promoter transgenes direct macrophage-lineage expression in zebrafish. *Blood* **117**, e49-56.

Langenau, D. M., Ferrando, A. A., Traver, D., Kutok, J. L., Hezel, J.-P. D., Kanki, J. P., Zon, L. I., Look, A. T. and Trede, N. S. (2004). In vivo tracking of T cell development, ablation, and engraftment in transgenic zebrafish. *Proc. Natl. Acad. Sci. USA* **101**, 7369–7374.

Walters, K. B., Green, J. M., Surfus, J. C., Yoo, S. K. and Huttenlocher, A. (2010). Live imaging of neutrophil motility in a zebrafish model of WHIM syndrome. *Blood* **116**, 2803–2811.
